# What should be the protocol selection after failure of in-vitro fertilization at normoresponder patients: Agonist or antagonist?

**DOI:** 10.4274/tjod.03789

**Published:** 2014-12-15

**Authors:** Seyit Temel Ceyhan, Yeşim Bayoğlu Tekin, Mehmet Sakinci, Cihangir Mutlu Ercan, Uğur Keskin

**Affiliations:** 1 Gülhane Military Medical Academy, In-Vitro Fertilization Unite, Ankara, Turkey; 2 Recep Tayyip Erdoğan University Faculty of Medicine, Department of Gynecology and Obstetrics, Rize, Turkey; 3 Akdeniz University Faculty of Medicine, Department of Gynecology and Obstetrics, Antalya, Turkey

**Keywords:** ovulation induction, GnRH agonist, GnRH antagonist, in vitro fertilization

## Abstract

**Objective:**

Evaluation of the impact of agonist or antagonist protocol selection on pregnancy outcomes after failure of in-vitro fertilization (IVF) treatment cycles which were down regulated with Gonadotropin Releasing Hormone (GnRH) agonist.

**Materials and Methods:**

This was a retrospective study. Two hundred and sixty nine patients who were treated with GnRH agonist protocol between years 2002-2012 at an IVF unit and underwent a second attempt following one year period after failure of IVF enrolled in the study. Age, basal FSH levels, antral follicle counts, duration of induction, the number of yielded oocytes, the number of transferred embryos and the transfer days, clinical and ongoing pregnancy rates were evaluated for each treatment cycle.

**Results:**

Normoresponder patients were separated into two groups according to the agonist or antagonist protocol selection at the second attempt and the results of two consequent IVF cycles were compared. There were no statistically significant difference between the groups for the dosage of administered gonadotropin, duration of induction, the count of yielded oocytes, the day and the number of transferred embryos (p>0.05). Furthermore the fertilization rate, clinical and ongoing pregnancy rates were similar in two groups.

**Conclusion:**

The selection of antagonist treatment is effective as agonist protocols at normoresponder patients after failure of IVF.

## INTRODUCTION

Thirty-five years having passed after the first successful in-vitro fertilization (IVF), it has become a commonly used method in many infertile patient groups. In the mid 1980’s, Gonadotropin Releasing Hormone (GnRH) agonists took their place in IVF treatment. GnRH agonists are used to suppress the pituitary positive feedback, which develops due to supra-physiologic estradiol levels caused by multi-follicular development^(1)^. In this way, premature Luteinizing Hormone (LH) peak, premature luteinizing and ovulation are prevented.

Subsequently, GnRH antagonists have entered into clinical use. Unlike GnRH agonists, GnRH antagonists provide immediate cessation of gonadotropin production by competitive receptor blockage^(2)^. Administrations of GnRH antagonists are limited to mid and late follicular phases. GnRH antagonists are generally preferred in IVF treatment in patients with low ovary reserve or in those on whom previous agonist protocols have been attempted and follicular development was poor. The most important reason for GnRH antagonists not having been preferred in the primary treatment is the published meta-analyses that report lower pregnancy rates^(3,4)^. There are insufficient number of studies in the literature comparing the effect of GnRH antagonist treatment on IVF success in young and the normoresponder patient group.

In our study, we aimed to compare the cycle results of the young and normoresponder patients in whom ovulation induction was performed by down regulating with GnRH agonists; however, IVF treatment has resulted in failure and again, down-regulation with GnRH agonist was preferred in the following cycles and in patients in whom the treatment protocol was replaced with GnRH antagonist.

## MATERIALS AND METHODS

This was a retrospective study. Medical records of the patients who were applied Assisted Reproductive Techniques (ART) between 2002 and 2012 in Gülhane Military Medical Academy (GATA) hospital’s IVF unit were evaluated. Two hundred and sixty nine women who had IVF failure at the first attempt with administration one of the luteal long or short protocols of GnRH agonists and taken into the treatment cycle again within one year were enrolled in the study. Approval was obtained from the Gülhane Military Medical Academy Ethics Committee for the study.

The patients undergoing IVF due to unexplained infertility, tubal factors, oligo-anovulation and secondary infertility were included in the study. The patients over thirty five years of age, the male factor (since this would affect the fertilization rates) (severe oligoasthenoteratosoospermia (total motile sperm number of <5 million) or azoospermia), severe endometriosis or decreased ovary reserve (According to the Bologna criteria: 1-Advanced maternal age (≥40 years) or presence of other risk factors for a poor ovarian response, 2- Previous history of poor ovarian response (collection of less than three oocytes with conventional ovarian hyper stimulation methods), 3- Abnormal ovarian reserve test FSH>10 IU/mL; presence of at least two of them(5)) were excluded from the study. Normo-responder patient criteria were accepted as; <35 years, presence of 5-9 antral follicles in both ovaries, not having a history of a poor response and the patients in whom at least 4 follicle developments had been detected after ovarian hyper-stimulation.

Furthermore, the patients with cancelled controlled ovarian hyper-stimulation (COH) because of poor ovarian response during the treatment or those from whom oocytes could not be collected during oocyte pick-up (OPU) process, total fertilization failure or patients in whom embryo transfer could not be accomplished due to lack of development of embryos, were also excluded from the study. 

Recombinant or urinary gonadotropin 150-450 IU was administered to cases during the first treatment cycles together with the luteal long or short agonist protocol, following the leading of at least 3 follicles reaching 17 mm, and oocyte maturation was triggered by application of 10 000 IU human chorionic gonadotropin (hCG). Thirty-six ours after hCG administration, the OPU procedure was performed under sedo-analgesia.

After having incubated the collected oocytes for 2 hours, enzymatic denudation with 80 IU of hyaluronidase and then, the mechanic denudation process were performed. Ejaculated sperms were prepared by using the standard gradient method (Isolate, Irvine Scientiﬁc, Santa Ana, California USA). Micro-injection was administered to all patients. Embryo developments were observed after fertilization and embryo transfer was performed on the 3^rd^ or 5^th^ days. If βhCG were negative or the pregnancy would result with chemical pregnancy or abortion 14 days after the transfer process, it was accepted as IVF failure. Clinical pregnancy diagnosis was made by ultrasonography by visualizing the gestational sac and observation of the fetal heart beats.

A comparison was made between the patients who had undergone one year of the first treatment cycles and down regulation with GnRH agonists in the second treatment cycles, and patients in whom ovulation induction had been applied with gonadotropin and who used the GnRH antagonist protocol. The protocol selection in the second cycles was left to the clinician’s choice and there were no patient selection criteria used. In the GnRH antagonist protocol, ovulation induction was initiated on the second day of the menstrual cycles with 150-450 IU of recombinant or urinary gonadotropin and then on the 6th day of the cycles, 0.25 mg Cetrotide (Cetrorelix, Serono, Turkey) was initiated and continued until the hCG day.

Statistical analysis was performed using the SPSS version 17.0. The patients were divided into two groups as patients using the agonist or the antagonist in the second cycles. The data were given as mean ± standard deviation. The normality distribution of the data was evaluated with the Lilliefors test. The number of the oocytes collected during the GnRH agonist and GnRH antagonist treatment cycles, the metaphase II (MII) oocyte number, the embryo transfer day, the number of transferred embryos, the applied gonadotropin amount and induction durations were compared. The Student’s t test was used for the comparison of the parametric values; the Wilcoxon analysis was used for the comparison of the non-parametric values, and the Chi-square test was used for the comparison of the nominal values. A p value of <0.05 was accepted as significant.

## RESULTS

A total of 269 patients in whom consecutive IVFs were applied twice within one year, were evaluated. Two hundred thirty-two patients (86.2%) had received the agonist protocol in the second cycles and 37 (13.8%) had received the antagonist protocol. The causes of infertility were: Unexplained infertility in 194 (72.1%), tubal factors in 60 (22.3%), oligo-anovulation in 10 (3.7%), and 5 (1.9%) were identified as secondary infertility. There was no statistically significant difference observed between the groups in terms of infertility causes (p=0.302). The distributions of the treatment protocols in the primary and secondary cycles have been demonstrated in Table 1. There was a significant difference between the two cycles with regard to the treatment choice (p<0.05).

The demographic data of the groups in which administered GnRH agonist and GnRH antagonist including: The mean age, body mass index (BMI), antral follicle numbers and basal FSH levels have been demonstrated in Table 2. According to this table, there was no statistically significant difference between the demographic characteristics of the patients. The patients receiving GnRH agonist in the second cycle were compared with antagonist-receiving patients according to their first and second cycle outcomes and given in Table 3. There was no statistically significant difference between the GnRH antagonist-receiving patients’ cycles results with previous cycles and the GnRH agonist-receiving patients’ results (p>0.05).

The fertilization rates, the pregnancy rate per embryo transfer, the clinic pregnancy rates and ongoing pregnancy rates of the GnRH agonist down-regulation applied patients with GnRH antagonist protocol applied women were presented in Table 4. There were similar fertilization rates, pregnancy rate per embryo transfer, clinical pregnancy and ongoing pregnancy rates in both treatment protocols. There was no statistically significant difference determined between the groups (p>0.05).

## DISCUSSION

We have compared the effects of the selected treatment protocols on treatment results in normoresponder patients in whom the first IVF cycle had resulted with failure and who underwent a second treatment applied within one year. The treatment results of the GnRH antagonist-receiving normoresponder patients were seen to be similar when compared with the patients’ previous cycles and the GnRH agonist protocols-receiving patients.

GnRH antagonists have entered ART practices as patient-friendly IVF applications^(6)^. When compared with GnRH agonists, the advantages of the GnRH antagonists include: Shortening the treatment duration in ovulation induction applications, decreasing the used exogenous gonadotropin amount, decreasing the frequency of hypo-estrogenic symptoms, decreasing the risk of functional cyst development risk, decreasing the incidence of ovarian hyper-stimulation syndrome (OHSS)^(7)^. However, as in the use of GnRH agonists, it is not possible in antagonist cycles to program the ovarian stimulation cycle previously, and decrease in pregnancy rates have been reported in the comparative studies^(8,9)^. Evaluation of the IVF success with pregnancy rates have influenced the stance of the clinicians for antagonist treatments and have rendered its preference generally in patients with poor response or previous unsuccessful cycles^(10,11)^.

However, when GnRH antagonists are used in patients with similar demographic characteristics, they reach equal pregnancy rates as with GnRH agonist protocols^(12)^. In a retrospective cohort study conducted by Johnston-MacAnanny et al., GnRH agonist with GnRH antagonist treatments were compared in normoresponder patients in the first cycle and there was no significant difference determined in both groups statistically in terms of the implantation rate, clinical pregnancy and live births^(13)^.

In the meta-analysis of Al-Inany et al., including 45 randomized controlled studies (RCS), there was no significant difference reported for ongoing pregnancy (28 RCS; OR 0.87, 95% CI 0.77-1.00) and live birth rates (9 RCS; odds ratio (OR) 0.86, 95% CI 0.69 -1.08) when GnRH antagonists were compared with the luteal long protocol^(14)^. In the meta-analysis performed by Pu et al., when the collected oocyte numbers were compared with the obtained mature oocyte numbers, it was demonstrated that there was no significant difference between the agonist and the antagonist receiving groups statistically^(15)^.

In the randomized controlled study conducted by Lainas et al. in women with polycystic ovary syndrome (PCOS), the antagonist protocol was compared with the luteal long protocol and in terms of continuing pregnancy rates, there was no significant difference between the two groups; however, the stimulation duration (10 vs 12 days difference is 2 days, 95% CI: +1, +2, p<0.001) and the used gonadotropin amount (1575 vs 1850 IU, difference-275 IU, 95% CI:- 25, -400, p<0.05) was demonstrated to be significantly lower in antagonist protocol used patients^(16)^. Similarly, in the study of Onofriescu et al., in which they compared the antagonist protocol with the luteal long agonist protocol in cases with PCOS, it was reported that the antagonist protocol provided less OHSS risk, a shorter stimulation period, a smaller degree of gonadotropin use and similar clinical and ongoing pregnancy rates^(17)^.

Felberbaum et al. have compared GnRH antagonist treatments with luteal long and agonist GnRH protocols in the group under 35 years of age, in the first treatment cycle, having only tubal infertility and classical IVF-applied patients which they classified as the ideal patient group and reported similar embryo transfer rates and clinical pregnancy rates in antagonist-applied patients similar to other agonist protocols^(18)^. Gordts et al. have compared the short agonist protocol with the antagonist protocol and reported similar implantation, ongoing pregnancy and live birth rates, and also similar cycle periods and obtained oocyte rates^(19)^.

In the randomized controlled study conducted by Moraloğlu et al., GnRH antagonists and luteal long agonist-receiving normoresponder patients were compared^(20)^. In this study, despite the longer lasting cycles and more follicle development in the agonist group, there was no statistically significant difference observed in terms of the obtained oocyte numbers, the developed and transferred embryo numbers and the fertilization rates. In the retrospective study conducted by Çelik et al., similar to our study, the results of the IVF cycles were compared in agonist and antagonist-receiving normoresponder patients and similar pregnancy rates were demonstrated^(21)^. Again, in a study conducted in Turkey, the similar normoresponder patient group received consecutive luteal long agonist and antagonist protocols, and shorter induction durations and higher implantation rates were determined in the antagonist-receiving cycles, and it was reported that antagonist treatment was as effective as luteal long agonist treatment in the normoresponder patient group^(22)^.

In this study, comparison of two consequent cycle results in the same patient group and calculation of the clinical and ongoing pregnancy rates were the dimensions that will contribute to the literature and which is different from other studies. However, one of the limitations of our study was that it was a retrospectively designed study. Randomized prospective designed studies with similar demographic characteristics and numbers of the groups are needed with regard to this subject. Determination of the treatment protocol by the clinician is a risk for bias. IVF-applied patients are mostly referred from outer institutions and follow-up of the pregnancies cannot be conducted in our center, which are limiting factors in terms of a healthy determination of ongoing pregnancy rates.

Consequently, when GnRH antagonist treatment is compared with GnRH agonist protocols, the results of fertilization, implantation and pregnancy rates are observed to be at similar levels. Within the scope of the current knowledge, in agonist protocols-receiving normoresponder patients, the GnRH antagonist protocol following the treatment cycles after IVF failure can be an appropriate alternative. GnRH antagonists are an effective choice against GnRH agonists, due to the lower number of side effects, lower complication rates and lower amount of used medicine besides not affecting the pregnancy rates. However, more comprehensive studies including more patients and groups that are matched are needed regarding this subject.

## Figures and Tables

**Table 1:**
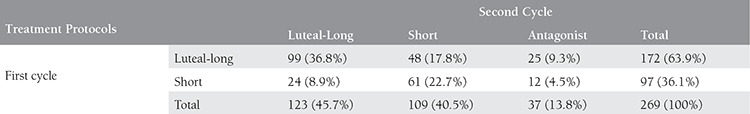
Distribution of treatment protocols according to IVF cycles

**Table 2:**
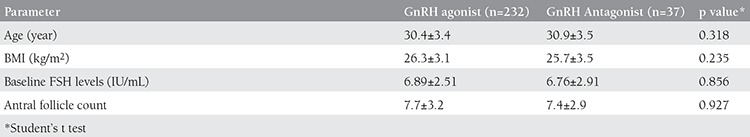
Comparison of the demographic characteristics of GnRH agonist and GnRH antagonist groups

**Table 3:**
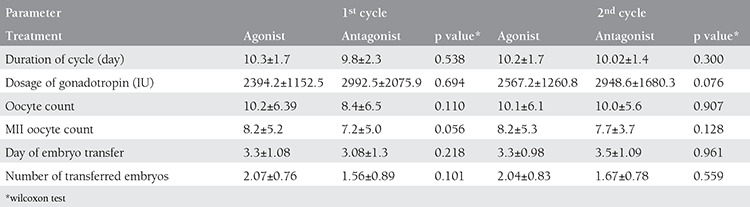
Comparison of both cycle outcomes of the patients who were administered GnRH agonist or antagonist at the second attempt

**Table 4:**
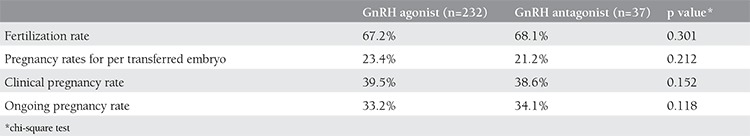
Comparison of pregnancy outcomes of GnRH agonist and GnRH antagonist groups

## References

[1] Albano C, Felberbaum RE, Smitz J, Riethmüller-Winzen H, Engel J, Diedrich K, et al (2000). Ovarian stimulation with HMG: results of a prospective randomized phase III European study comparing the luteinizing hormone-releasing hormone (LHRH)-antagonist cetrorelix and the LHRH-agonist buserelin. European Cetrorelix Study Group. Hum Reprod.

[2] Felberbaum RE, Reissmann T, Küpker W, Bauer O, al Hasani S, Diedrich C, et al (1995). Preserved pituitary response under ovarian stimulation with HMG and GnRH antagonists (Cetrorelix) in women with tubal infertility. Eur J Obstet Gynecol Reprod Biol.

[3] Kolibianakis EM, Tarlatzis B, Devroey P (2005). GnRH antagonists in IVF. Reprod Biomed Online.

[4] Ludwig M, Katalinic A, Diedrich K (2001). Use of GnRH antagonists in ovarian stimulation for assisted reproductive technologies compared to the long protocol Meta-analysis. Arch Gynecol Obstet.

[5] Ferraretti AP, La Marca A, Fauser BC, Tarlatzis B, Nargund G, Gianaroli L, et al (2011). ESHRE consensus on the definition of ‘poor response’ to ovarian stimulation for in vitro fertilization: the Bologna criteria. Hum Reprod.

[6] Olivennes F, Frydman R (1998). Friendly IVF: the way of the future?. Hum Reprod.

[7] Depalo R, Jayakrishan K, Garruti G, Tataro I, Panzarino M, Giorgino F, et al (2012). GnRH agonist versus GnRH antagonist in in vitro fertilization and embryo transfer (IVF/ET). Reprod Biol Endocrinol.

[8] Al-lnany H, Aboulghar M (2001). Gonadotiophin-releasing hormone antagonists for assisted conception. Cochrane Database Syst Rev 2001;.

[9] Fauser BC, Devroey P (2005). Why is the clinical acceptance of gonadotropin releasing hormone antagonist co-treatment during ovarian hyperstimulation for in vitro fertilization so slow?. Fertil Steril.

[10] Griesinger G, Felberbaum R, Diedrich K (2005). GnRH-antagonists in reproductive medicine. Arch Gynecol Obstet.

[11] Griesinger G, Felberbaum R, Diedrich K (2005). GnRH antagonists in ovarian stimulation: a treatment regimen of clinicians’ second choice? Data from the German national IVF registry. Hum Reprod.

[12] Engel JB, Griesinger G, Schultze-Mosgau A, Felberbaum R, Diedrich K (2006). GnRH agonists and antagonists in assisted reproduction: pregnancy rate. Reprod Biomed Online.

[13] Johnston-MacAnanny EB, DiLuigi AJ, Engmann LL, Maier DB, Benadiva CA, Nulsen JC (2011). Selection of first in vitro fertilization cycle stimulation protocol for good prognosis patients: gonadotropin releasing hormone antagonist versus agonist protocols. J Reprod Med.

[14] Al-Inany HG, Youssef MA, Aboulghar M, Broekmans F, Sterrenburg M, Smit J, et al (2011). Gonadotrophin-releasing hormone antagonists for assisted reproductive technology. Cochrane Database Syst Rev.

[15] Pu D, Wu J, Liu J (2011). Comparisons of GnRH antagonist versus GnRH agonist protocol in poor ovarian responders undergoing IVF. Hum. Reprod.

[16] Lainas TG, Sfontouris IA, Zorzovilis IZ, Petsas GK, Lainas GT, Alexopoulou E, et al (2010). Flexible GnRH antagonist protocol versus GnRH agonist long protocol in patients with polycystic ovary syndrome treated for IVF: a prospective randomized controlled trial (RCT). Hum Reprod.

[17] Onofriescu A, Bors A, Luca A, Holicov M, Onofriescu M, Vulpoi C (2013). GnRH Antagonist IVF Protocol in PCOS. Curr Health Sci J.

[18] Felberbaum R, Griesinger G, Finas D, Dawson A, Diedrich K (2005). To agonize or antagonize in gonadotrophin stimulation cycles?. Reprod Biomed Online.

[19] Gordts S, Van Turnhout C, Campo R, Puttemans P, Valkenburg M, Gordts S (2012). A prospective randomised study comparing a GnRH-antagonist versus a GnRH-agonist short protocol for ovarian stimulation in patients referred for IVF. Facts Views Vis Obgyn.

[20] Moraloglu Ö, Kilic S, Karayalçin R, Yuksel B, Tasdemir N, Işik A, Ugur M (2008). Comparison of GnRH Agonists and Antagonists in Normoresponder IVF/ICSI in Turkish Female Patients. Adv Ther.

[21] Çelik S, Gürbüz B, Cengiz Çelik C, Purisa S (2013). Kontrollü Ovarian Hiperstimulasyon/İntrastoplazmik Sperm Enjeksiyonu Yapılan Normal Cevaplı Kadınlarda Uzun ve Antagonist Protokollerinin Gebelik Sonuçlarına Etkisinin Karşılaştırılması. İstanbul Med J.

[22] Kumbak Aygun B, Kahraman S (2010). Comparison of GnRH Agonist Long and Antagonist Protocols in the Same Normoresponder Patient Undergoing Assisted Reproductive Treatment. Fırat Tıp Dergisi.

